# Cross-cultural adaptation and validation of the Turkish version of the sinus and nasal quality of life survey (SN-5)

**DOI:** 10.14744/nci.2021.94547

**Published:** 2022-03-24

**Authors:** Berkay Caytemel, Can Doruk, Levent Aydemir, Hakan Kara, Senol Comoglu, Meryem Nesil Keles Turel

**Affiliations:** Department of Otolaryngology & Head and Neck Surgery, Istanbul University, Istanbul Faculty of Medicine, Istanbul, Turkey

**Keywords:** Pediatrics, quality of life, rhinitis, sinusitis, Turkish, validation studies

## Abstract

**Objective:**

The Sinus and Nasal Quality of Life (QoL) Survey (SN-5) is a valid questionnaire that evaluates the QoL of the pediatric population associated with sinonasal diseases and symptoms. The aims of this study were to translate the SN-5 test to Turkish language (SN-5t), evaluate the internal consistency of the test and test-retest reliability and validate the translation for further use in studies in Turkish language.

**Methods:**

In this prospective study, 50 healthy subjects and 50 patients, age between 2 and 12, with sinonasal symptoms prolonged over 1 month were included to the study. Families of healthy subjects were asked to fill the SN-5t twice with 1-week interval. The patient group completed test once prior the treatment and once 4 weeks after the treatment. Cronbach’s test was performed to test internal consistency and Spearman’s test was performed to evaluate test-retest validity.

**Results:**

The median value of the pre-treatment tests of the patient group and control group was 25 (23–28) and 14.25 (12–16), respectively. A statistically significant difference was found between groups (p<0.001). Area under the receiver operating characteristics (ROC) curve (A_roc_) value was calculated as 0.992 which stated the strong diagnostic accuracy, and the cutoff point was defined as 16.5. Cronbach’s alpha value of 0.75 was found. The Spearman’s rank correlation coefficient value (Spearman’s rho) was calculated as 0.946.

**Conclusion:**

The Turkish translation of the SN-5 is a consistent and valid test with high sensitivity and specificity that can be used in studies including Turkish speaking population.

**S**inonasal symptoms are major causes of outpatient health-care application and health-care costs throughout the world. Chronic and recurrent nasal symptoms affect approximately 10% of the population worldwide [1]. Like the adult population, sinonasal symptoms are also very common in pediatric patients, of which mostly caused by allergic rhinitis [2]. Sinonasal diseases deteriorate the quality of life (QoL) of children, and one of the main aim of the treatment is to increase the QoL.

The World Health Organization defined health as a complete physical, mental and social wellness which leads to an increase in research on disease-specific surveys that evaluates physical, mental, and social impacts of diseases on patients’ QoL [3]. Many surveys have been developed to evaluate the effects of sinonasal diseases such as the Sino-Nasal Outcome Test-20 (SNOT-20)[4] and SNOT-22 [5] and Nasal Obstruction Symptom Evaluation scale (NOSE) [6].

The Sinus and Nasal QoL Survey (SN-5) has been created and validated [7] to evaluate the impacts of sinonasal symptoms on pediatric patients’ QoL efficiently and easily. The test evaluates the effects of disease on physical, emotional, social and overall wellness of patients for the previous 4 weeks.

The aim of this study was to translate SN-5 to Turkish language, evaluate the internal consistency of the test, test-retest reliability, and validate the adaptation for further use in studies in Turkish language.

## Materials and Methods

This prospective study has been completed in our Otolaryngology and Head and Neck Department between May and December 2019. Ethics Committee Approval for the study was taken from our hospital’s Istanbul University Faculty of Medicine Ethics Committee for Scientific Research with file number 2019/723. Approval from the creator of the SN-5 test has been taken.

### Questionnaire

SN-5 has been created for pediatric population, thus instead of the patients, families of the patients answered the questions. SN-5 test consists of 5 questions that evaluate the degree of sinus infection, nasal obstruction, allergy symptoms, emotional distress, and activity limitations of the patients by only taking into account of the past 4 weeks. The participants require to choose the best answer that reflects the degree of symptoms out of 7 possible answers. Afterward, a numerical scale is crated for the answers; the patient gets one point if there is no symptom present, 7 for symptoms that cause trouble at all time. After completing the five questions, participants are asked to mark a Visual Analog Scale (VAS) that evaluates the sinonasal specific overall QoL.

### Translation

**Figure 1.**
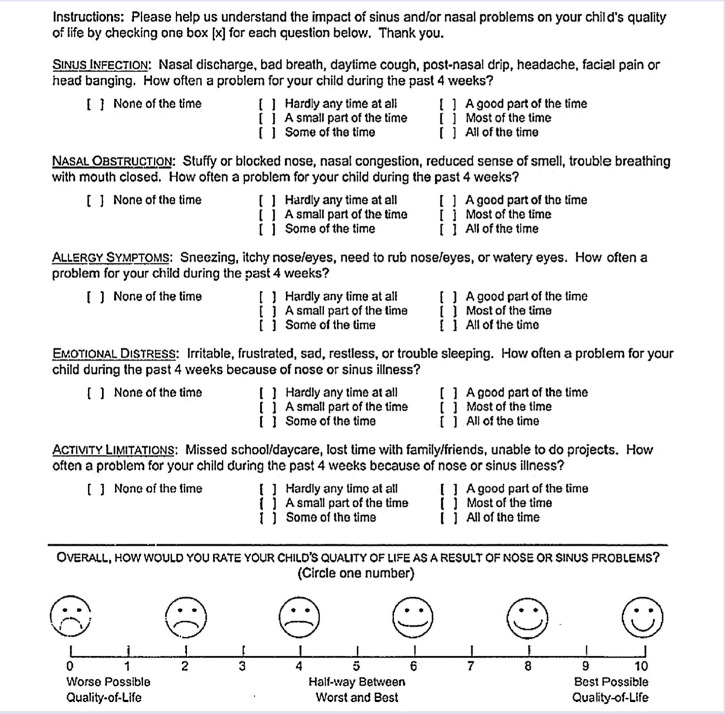
Original questionnaire of SN-5 (English).

**Figure 2.**
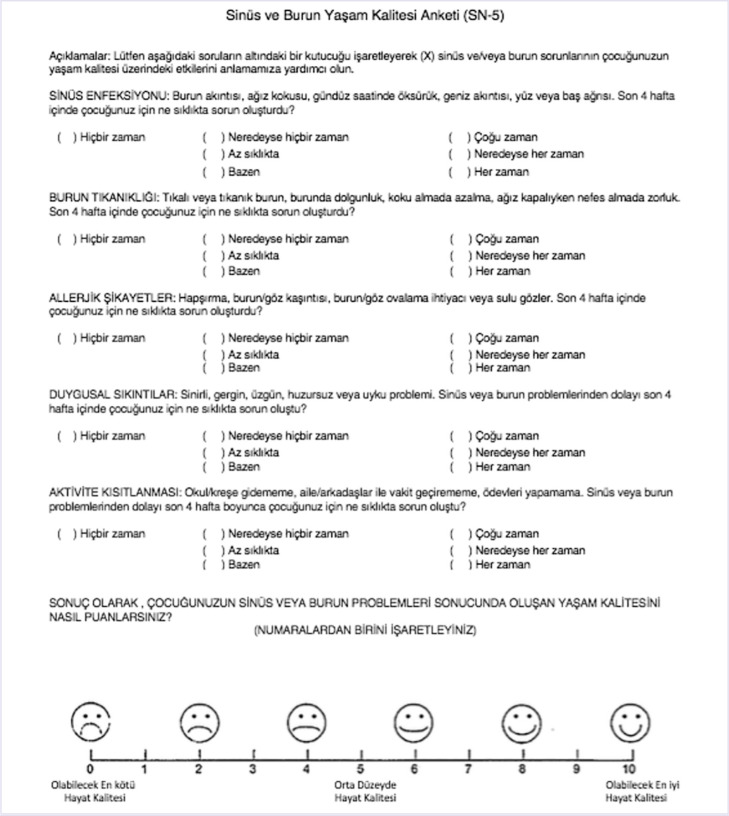
Turkish version of SN-5 Questionnaire.

We followed the recommendations of the WHO for the translation and adaptation of instruments:

1. The original version of SN-5 (Fig. 1) was translated by two otolaryngologists and two rhinologists (v1).
2. v1 was sent to a panel of three experts in otolaryngology (different from the first translators). Two versions of the translated test (v2 and v3) were developed until a general consensus was reached.
3. The third version (v3) was sent to a native English-Turkish speaker for back-translation. No changes were made.
4. Afterwards, a pre-test cognitive interview was carried out in 20 patients to check for comprehensibility in the translated questionnaire (Fig. 2). There were no changes after this phase either.

**Highlight key points**

• The Sinus and Nasal QoL Survey (SN-5) has been created and validated to evaluate the impacts of sinonasal symptoms on pediatric patients’ QoL efficiently and easily.e
• There is no validated Turkish questionnaire measuring the QoL associated with sinonasal symptoms in pediatric patients.
• This is the first study which translates SN-5 to Turkish language, evaluate the internal consistency of the test, test-retest reliability, and validate the adaptation for further use in studies in Turkish language.

### Subjects

Informed written consents from the caregivers (at least two adults) of the patients before filling the questionnaire were taken.

A total of 100 pediatric patients between 2 and 12 years of age were included to study. Patient group consists of 50 volunteers who have sinonasal symptoms over a period of 4 weeks such as nasal or postnasal discharge, nasal obstruction, nasal fullness, daytime cough, and bad breath. The control group was selected from patients who applied to our clinic for routine otolaryngologic check-up. The control group consists of 50 volunteer pediatric subjects that do not have any sinonasal symptoms and pathologies in physical examination.

Exclusion criteria of the study includes; (a) caregiver of patients who are not able to give informed consent, (b) caregivers of patients who are not native Turkish speakers, (c) patients who are diagnosed with obstructive sleep apnea due to adenotonsillar hypertrophy, (d) developmental problems and/or craniofacial anomalies, and (e) patients who are diagnosed with diseases that might cause secondary chronic rhinosinusitis.

SN-5 test was completed twice by both of the groups. Caregivers of the patients completed the questionnaire once at the time of the first visit (Group 1a) and once after 4 weeks of standard treatment (Group 1b). Caregivers of the control group completed the questionnaire once at the time of the visit (Group 2a) and once 1 week after the initial test via phone call or e-mail (Group 2b). The “standard treatment” includes nasal irrigations and nasal steroids, and for patients with purulent anterior/posterior drainage or fever, addition of oral antibiotics [8, 9].

### Statistical Analysis

Power analysis was performed with PASS 11 (Power analysis and sample size software, NCSS, LLC, Kaysville UT, USA, ncss.com/software/pass). To detect the difference between the coefficient alpha under the null hypothesis of 0.3 and the alternative hypothesis of 0.7 using a two-sided F-test with a power of 0.95 and an α of 0.05, required patient number was 47, each responding to five items. Considering the possible drop-outs, we decided to involve 50 patients.

Statistical Package for the Social Sciences (SPSS) version 22 (IBM Corp., Armonk, NY, USA) was used to compute other statistical tests. A value of p<0.05 was considered as statistically significant.

The Shapiro–Wilk test was performed to evaluate the normality of data distribution. P values of overall scores of the initial tests for Group 1a and Group 2a were 0.331 and 0.005, respectively. Due to abnormal distribution of Group 2a, non-parametric tests have been chosen for further analysis. Mann-Whitney U test was performed for the validation of the questionnaire by comparing the overall scores of the Group 1a and 2a. To evaluate the questionnaire’s ability for assessing treatment outcomes, Wilcoxon signed-rank test was used.

A receiver operating characteristics (ROC) curve was drawn and the area under the ROC curve (A_roc_) was calculated to show diagnostic value of the SN-5 questionnaire.

Cronbach’s alpha was calculated to estimate the internal consistency and the inter-item correlation of the questionnaire. A coefficient of 0.70 or higher was acceptable [10].

The test-retest reliability was assessed by measuring spearman’s rank correlation coefficient value (Spearman’s rho). A value of >0.7 is generally acceptable in the literature [11].

### Ethical Standards

The study obtained the Approval of the Ethical Committee of our Hospital in accordance with the Declaration of Helsinki. All parents or legal guardians of the included participants provided written consent information.

## Results

Demographic information of the control and patient groups is given in Table 1.

**Table 1.** Demographic information of the patient and control groups

Age	Patient group (n=50)	Control group (n=50)
Mean	6.44	6.306
SD	2.733	2.778
Minimum	2	2
Maximum	12	12
Sex		
Male	31	27
Female	19	23

n: Number; SD: Standard deviation.

The median [First to third quartile] overall score of SN-5 for group 1a (25 [23–28]) was significantly higher than for Group 2a (14,25 [12–16]) (p<0.001).

After the application of recommended treatments 1 month later; the median overall score of second test (Group 1b) was 15 (11.75–16) (p<0.001), and significant improvement was observed in all symptoms (p<0.001) (Table 2).

**Table 2.** On initial and final assessment; median and interquartile range sinonasal complaint scores

Sn-5 Questionnaire	Initial*	Post-treatment*	p
Sinus infection	5.5 (5–6)	3 (3–4)	<0.001
Nasal obstruction	6 (5–6)	3 (2–3)	<0.001
Allergy symptoms	5 (5–6)	3 (2–3)	<0.001
Emotional distress	5 (4–6)	3 (2–3)	<0.001
Activity limitations	4 (3.75–5)	3 (2–3)	<0.001
Overall score	25 (23–28)	15 (11.75–16)	<0.001
VAS Score	4 (3–4)	7 (7–8)	<0.001

Sn-5=Sinus and Nasal Quality of Life Survey; VAS: Visual Analog Scale; *: Data were presented as median (first to third quartile).

**Figure 3.**
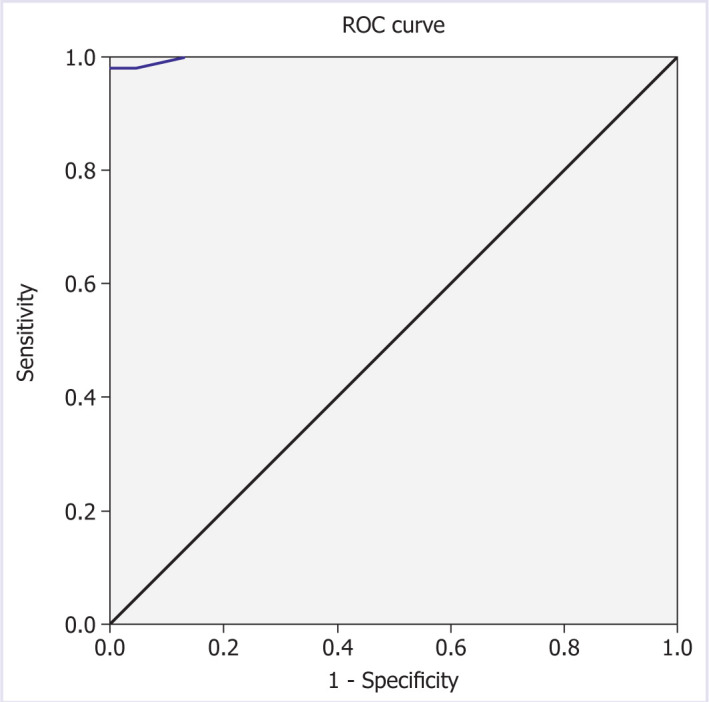
The questionnaire receiver operating characteristics (ROC) curve. A^roc^ value was measured as 0.998 and a cutoff point was 16.5.

The ROC curve was drawn (Fig. 3). A_roc_ value was measured as 0.998, and a cut offpoint of 16.5 was selected.

The Cronbach’s alpha was 0.75. Thus, the inter-item correlation and the internal consistency of the questionnaire were adequate.

Test-retest reliability 1 week after the initial test-results between group 2a-2b-was significantly high (r=0.946, p<0.001).

**Figure 4.**
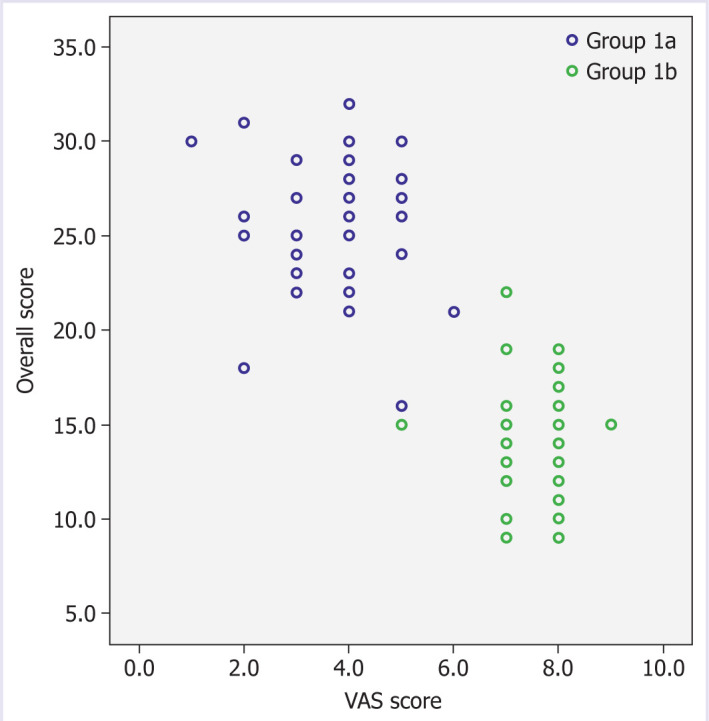
Scatter plot. VAS, visual analog scale.

The overall SN-5 score of both Group 1a and 1b was inversely correlated with the VAS score (r=0.827, p<0.001) (Fig. 4).

## Discussion

The increased life expectancy of the human population causes and upraise in the incidence of chronic diseases, which highlights the significance of the patient-based evaluation of the symptoms and targeting the treatment according to the symptoms. To achieve this, use of disease specific QoL measures is a common method that gives the opportunity to evaluate and compare the treatment methods in terms of efficacy and adverse effects. Furthermore, disease specific QOL measures are developed specific for the disease, which increases the internal consistency, sensitivity and specificity of the measure [3].

There are serious differences between patient’s clinical and endoscopic examination findings and complaints, especially in rhinology. In many sinonasal diseases, there is no relationship between computed tomographic findings and symptom severity, which is accepted as the most valuable imaging method in sinonasal diseases. For these reasons, QoL scales can help us to determine the severity of sinonasal diseases [12]. Many studies have been developed and used in clinic to evaluate the effects of sinonasal diseases on adults such as Sino-Nasal Outcome Test-20 (SNOT-20) [4] and SNOT-22 [5] and NOSE [6].

Studies on pediatric QoL studies show that in order to prevent adulthood health problems related to childhood diseases use of QoL measurements might be useful; even though applying QoL questionnaires in pediatric groups is less convenient than adults. However, evaluating the QoL in pediatric patients is often challenging. Parents have their own perceptions and perspectives on the QoL of their children. They may lose their objectivity in their responses to the clinician to convey that their child’s problems are major and important [13]. The first study to evaluate the overall QoL in pediatric patients was published by Eisen et al. in 1979 [14]. Then; various questionnaires have been developed to evaluate pediatric patients in terms of general health.

First QoL questionnaire in the field of otorhinolarngology for the pediatric population was created by Berdeaux et al. in 1999 [15]; which was designed as a general survey to evaluate the effects of ear, nose and throat diseases. Need of disease Specific QoL survey for sinonasal diseases has been fulfilled after development and validation of SN-5 survey [7]. SN-5 survey has been translated and validated to Portuguese before [16].

The validity of the study was evaluated by comparing Group 1a and Group 2a. The median overall score of SN-5 for Group 1a (25 [23–28]) was significantly higher than for group 2a (14.25 [12–16]) (p<0.001). These results show that Turkish version of SN-5 (SN-5t) is a valid test to be used in further studies.

Our results showed that there is a statistically significant difference between SN-5t values at the pre-treatment and post-treatment period (Group 1a and 1b) (p<0.001). Furthermore, significant improvement was observed in all symptoms (p<0.001), and SN-5 results of the both groups are inversely correlated with VAS scores (r=−0.827, p<0.001. Similar results have been indicated by Uchoa et al. [16] in the Portuguese adaptation and validation of the SN-5. These findings support that SN-5t can be used to demonstrate clinical improvement.

Our results showed an A_roc_ value of 0.998, and a cutoff point of 16.5 was selected. These findings showed very strong ability to differentiate sinonasal disease from healthy status, achieving the best-balanced sensitivity and specificity values (0.98 and 0.96, respectively).

The internal consistency of the survey was calculated using the Cronbach’s alpha value which was 0.75. This value was found 0.62 [7] in the original article and 0.73 in the Portuguese version [16] Our score was higher than 0.70 and close to 1, thus it can be stated that the inter-item correlation and internal consistency of the questionnaire is high [8].

The test-retest reliability of SN-5t was measured with Spearman’s rank correlation coefficient value. Our findings showed that the test-retest reliability of SN-5t is adequate (r=0.946, p<0.001). The result is also similar to Portuguese version (r=0.957, p<0.001) [16].

### Conclusion

SN-5t survey for the assessment of sinonasal diseases of the pediatric age is a valid test that can be used to evaluate the severity of the symptoms and degree of amelioration with the treatment. High sensitivity and specificity of the test shows that the survey can be used as a valuable item to evaluate pediatric patients with sinonasal symptoms.
